# Endocrine Disruptors and Autism Spectrum Disorder in Pregnancy: A Review and Evaluation of the Quality of the Epidemiological Evidence

**DOI:** 10.3390/children5120157

**Published:** 2018-11-23

**Authors:** Salvador Marí-Bauset, Carolina Donat-Vargas, Agustín Llópis-González, Amelia Marí-Sanchis, Isabel Peraita-Costa, Juan Llopis-Morales, María Morales-Suárez-Varela

**Affiliations:** 1 Unit of Public Health and Environmental Care, Department of Preventive Medicine, University of Valencia, Avenida Vicente Andrés Estellés s/n, Burjasot, 46100 Valencia, Spain; salvador.mari@ext.uv.es (S.M.-B.); agustin.llopis@uv.es (A.L.-G.); ivperaitacosta@hotmail.es (I.P.-C.); juanllopis6@gmail.com (J.L.-M.); 2 Nutritional Epidemiology, Institute of Environmental Medicine, Karolinska Institutet, Nobels väg 13, 171 65 Solna, Sweden; carolina.donat.vargas@ki.se; 3 Biomedical Research Center Network on Epidemiology and Public Health (CIBERESP), Institute of Health Carlos III, Avenida Monforte de Lemos, 3-5, Pabellón 11, Planta 0, 28029 Madrid, Spain; 4 Clinical Nutrition and Dietetics Unit, Navarra Hospital Complex, Calle de Irunlarrea, 3, Pamplona, 31008 Navarre, Spain; amarisanchis@gmail.com

**Keywords:** autism, ASD, child behavior disorders, endocrine disruptor, environmental exposure

## Abstract

Exposure to environmental contaminants during pregnancy has been linked to adverse health outcomes later in life. Notable among these pollutants are the endocrine disruptors chemicals (EDCs), which are ubiquitously present in the environment and they have been measured and quantified in the fetus. In this systematic review, our objective was to summarize the epidemiological research on the potential association between prenatal exposure to EDCs and Autism Spectrum Disorder (ASD) published from 2005 to 2016. The Navigation Guide Systematic Review Methodology was applied. A total of 17 studies met the inclusion criteria for this review, including: five cohorts and 12 case-control. According to the definitions specified in the Navigation Guide, we rated the quality of evidence for a relationship between prenatal exposure to EDCs and ASD as “moderate”. Although the studies generally showed a positive association between EDCs and ASD, after considering the strengths and limitations, we concluded that the overall strength of evidence supporting an association between prenatal exposure to EDCs and later ASD in humans remains “limited” and inconclusive. Further well-conducted prospective studies are warranted to clarify the role of EDCs on ASD development.

## 1. Introduction

Autism spectrum disorders (ASD) comprise a complex set of behaviorally defined neurodevelopmental abnormalities in two core areas: deficits in social communication and fixated, restricted, repetitive, or stereotyped behaviors and interests. Prevalence of ASD has significantly increased globally over the last few decades [1,2,3], and today it is estimated to be between 6.2 and 7.6 per 1000 persons [4,5]. ASD prevalence reported in the United States (US) reached 14.7 per 1000 (i.e., 1 in 68) in children aged eight years [6]. On the other hand, Christensen et al., (2016) describe ASD prevalence among four-year-old children in 5 of 11 sites participating in the 2010 Autism and Developmental Disabilities Monitoring Network as 13.4 per 1000, which was 30% lower than eight-year-old ASD prevalence, and the male-to-female ratio is nearly 5:1 [7,8].

### 1.1. Environmental Factors and ASD Causation

So far, the study of the etiology of ASD has focused mainly on identifying specific ASD risk genes [9,10]. Studies on the concordance of autism diagnosis between identical twins and among siblings have indicated a strong genetic component contributing to ASD [11,12]. Hallmayer et al., (2011) [13] in their study found that only 38% of ASD cases were attributable to genetic causes. However, studies examining the concordance rates between monozygotic twins revealed that although the concordance rate of ASD between monozygotic twins was higher than of dizygotic twins, the penetrance was still partial, revealing that genetic factors alone do not explain all of the pathogenicity and variability in ASD [14].

Findings like those aforementioned, together with the rapidly increasing prevalence of ASD, has led to recognize that environmental factors may play a significant role in the etiology and pathogenesis of ASD [15,16,17]. Gene-environment interactions during fetal development leading to early-life epigenetic changes are also known to affect subsequent gene expression in the brain [18], and to be behind the potential risk of ASD following the prenatal exposure to environmental factors [2,19,20,21,22,23,24,25,26,27]. Epigenetic modifications (the alteration of DNA transcription without alterations in the DNA sequence) affect gene regulation. These alterations in gene expression result from transcriptional regulatory influences of environmental factors, such as immunological effects, nutritional deficiencies, endocrine disruptors chemicals (EDCs), and pharmaceuticals [28].

### 1.2. EDCs and Human Exposure

Close to 800 chemicals are known or suspected to be capable of interfering the function(s) of the endocrine system. However, only a small fraction of these chemicals have been investigated are now beginning to be recognized as potential threats to health [29,30,31]. Some of these chemicals have the capacity to interfere with the endocrine system mimicking the action of endogenous hormones; antagonizing their mechanism of action; altering their pattern of synthesis, transport, release, or metabolism; or, by modulating the levels of the corresponding receptors [31]. The endocrine disruptor chemicals (EDCs) include a huge variety of human purposefully created chemicals with commercial, agricultural, industrial, or pharmaceutical applications. EDCs migrate into the air, food, and water of humans and wildlife. They are also incorporated into numerous products that are used in daily life (e.g., plastics, furniture, pesticides, cosmetics, drugs, household products, or even food), as well as in processes of combustion of fossil fuels, among others; and consequently, human exposure to EDCs may come from numerous sources.

They include bisphenol-A (BPA) and its structural analogs (e.g., BFS, BPF), phthalates, brominated flame retardants, perfluorate compounds, aromatic polycyclic hydrocarbons, dichlorodiphenyltrichloroethane (DDT), polychlorinated biphenyls (PCB), and some heavy metals [20,31,32,33,34,35], as summarized in Table 1. Their physicochemical properties, such as persistence, stability, and bioaccumulation capacity in the trophic chain varies greatly depending on the nature of compound. Thus, while some are very lipophilic, accumulate in the fatty tissue of living being and have long half-life (e.g., PCBs, dioxins, DDT), others are soluble in water (e.g., phthalates or biphenyl A), have a rapid excretion rate, and are not bioaccumulative (e.g., phthalates, BPA). However, low level exposure maintained over time to these hydrophilic EDCs, with less bioaccumulative potential and shorter half-life in the human bodies have been also associated with neurodevelopmental disorders [31,36,37].

### 1.3. Prenatal EDC Exposure and Risk for ASD

In 2012, the World Health Organization (WHO) and the United Nations Environment Programme (UNEP) defined EDCs as “exogenous substance or mixture that alters function(s) of the endocrine system and consequently causes adverse health effects in an intact organism, or its progeny, or (sub) populations” [31], expanding upon the concept of effects on subsequent generations (progeny).

Exposures in utero to even extremely low doses of EDCs during early development can alter sensitive biological processes, leading to permanent impairments in organ function [31,36,37,38,39,40,41,42]. The developing human brain is inherently much more susceptible to injury that is caused by toxic agents than is the brain of an adult. Specifically, the blood-brain barrier, which protects the adult brain from many toxic chemicals, is not completely formed until about six months after birth [43].

The most vulnerable and critical periods for toxic impact of pollutants on human development are the embryonic and fetal stages [44,45,46]. During fetal development, the placenta is not an effective barrier against most of the EDCs [47], which easily cross the placenta—around week 5 of embryo life, passing from the mother to the fetus [48]. EDCs concentrations in umbilical cord blood can be substantially higher than in maternal blood [49]. Moreover, the fetus has lower levels of several cytochrome P450 enzymes that metabolize environmental chemicals [50].

In ASD development, there is evidence that suggests than environmental exposures during these critical periods can permanently reprogram normal physiological responses (developmental reprogramming) in organogenesis and tissue differentiation [21,51,52,53,54,55]. Exposure to environmental chemicals during gestation has been associated with different neurodevelopmental disorders/deficits in children in both animal [56,57,58] and epidemiological studies [38,39,40,59,60,61,62,63,64].

The mechanisms by which EDCs act can range from gene expression to physiologic mechanisms, including steroid hormone receptor-mediated pathways. During pregnancy, the fetal brain has exquisite sensitivity to endogenous hormones from the mother and the fetus itself. These hormones, particularly steroid hormones, change the structure and function of the developing nervous system and their release is highly regulated, with very precise timing and levels needed to accomplish normal development [65]. The fact that ASD are approximately five times more prevalent in males than females has led some to propose a role of prenatal steroid hormone in the development of ASD [66]. Elevated fetal steroidogenic activity is associated with autistics traits [67,68]. It seems that the thyroid gland also play a key role in neurological fetal development [69,70].

Thus, bisphenol A and its structural analogs has been linked to reductions of thyroxine (T4) and thyroid stimulating hormone (TSH) levels [71]. Phthalates has also been associated with lower levels of fT3 and fT4 as well as progesterone [72]. Nevertheless, the relationships between hormones, neurodevelopment, and the autistic phenotype are not clear.

Several molecular mechanisms plausibly explain how long-lasting effects of prenatally EDCs could affect brain and behavior. These mechanisms usually go under the heading epigenetic. Skinner et al. (2008) [73], in animal models showed that embryonic and fetal exposure to environmental contaminants led to changes in the expression of several genes in the brain through epigenetic pathways, as DNA methylation, RNA-associated silencing, and histone modifications. There is however much to comprehend yet.

In light of the correlation over decades between increasing industrial chemical production and increasing rates of ASD diagnoses, to assess the current state of epidemiological evidence on prenatal EDCs exposure and ASD risk supporting the suggested biological plausibility is warranted.

## 2. Materials and Methods

A systematic review of the medical literature was performed to address and understand the potential association between EDCs exposure during pregnancy and ASD in offspring. The question was asked: Is pregnancy exposure to endocrine disrupting chemicals associated with increased risk of ASD development?

### 2.1. Search Strategy

The Navigation Guide Systematic Review Methodology [74,75]—adapted from Cochrane’s methodology and the Grading of Recommendations Assessment Development and Evaluation (GRADE) Working Group [76,77,78,79,80]—was applied for synthesizing the available scientific evidence and for rating the quality and strength of the evidence across all the retrieved studies. The Navigation Guide, which is based on the PECO statement [81,82], is a novel evidence-based medicine method for a systematic and transparent environmental health reviews. This approach assigns *a priori*, a “moderate” quality rating to observational studies based on the characteristics of the studies and considering adjustments (“downgrades” or “upgrades”). Ratings for each criteria range from −2 (2 level downgrade) to +2 (2 level upgrade) and 0 indicating no change from “moderate” quality. As described in Table 2, while five factors (i.e., risk of bias for each included study, inconsistence between studies, indirectness, imprecision, and likely publication bias) may lead to rating down the quality of evidence, other three factors (i.e., large effect size, all potential confounding factors, and existent dose-response gradient) lead to rating up.

We assessed risk of bias using as guidance the Cochrane Collaboration’s “Risk of Bias” tool and the Agency for Healthcare Research and Quality’s (AHRQ) criteria, which includes selection bias, confounding, performance bias, attrition bias, detection bias, and reporting bias. At the risk of bias tool (internal validity), we contemplated the following domains: recruitment strategy, blinding, confounding, exposure/outcome assessments, incomplete outcome/exposure data, selection bias, conflict of interest, and other bias [83].

### 2.2. Specify the Study Question

This approach is developed around a PECO (participants, exposure, comparator, and outcomes) statement. The PECO statement was the guide for the whole review process, including the definition of the research question, the bibliographic search strategy (i.e., search terms, inclusion/exclusion criteria), the quality and strength criteria, as well as the strategy for the synthesis and report of the results. Based on statement we included the following:

- *Participants*: pregnant women and their children of any age.
- *Exposure*: exposure to EDCs during pregnancy. The EDCs exposure was measured either through questionnaires/interviews held with parents, estimations provided by environmental agencies (Toxic Release Inventory (TRI), the US EPA National-scale Air Toxics Assessment (NATA), or analyses of biological samples.
- *Comparator*: works defined by ASD observational studies, and comparing the EDCs exposure levels for people with ASD versus those without.
- *Outcomes*: children of any age classified as having ASD disorder.

### 2.3. Study Identification and Eligibility Criteria

Following the Spanish National Health System recommendations, the search was based on Medline, although these other databases were also consulted: Cochrane Library, Scielo, Scopus, EMBASE, Google Scholar, PsychInfo, and Web of Science. Table 3 shows the search strategy using the following MeSH terms: Autism spectrum disorder, Autistic disorder, child behavior disorders, endocrine disruptors, environmental exposure, pesticides, pregnancy, prenatal, “in utero” with the corresponding Boolean operators.

**Figure 1:**
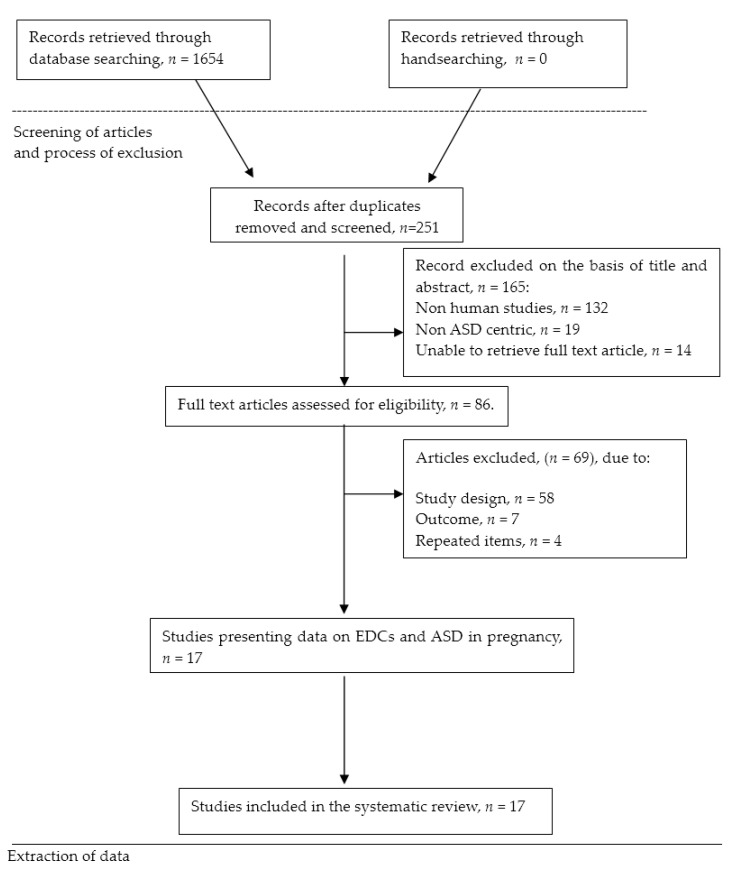
Flow chart of the systematic review process.

Figure 1 provides the flow chart for the study selection process, based on the PRISMA flow [84,85,86]. Original articles published from 2005 to date were initially retrieved. The last search was made on May, 2017. The 2005 cutoff date was considered to be appropriate because the increasing ASD incidence of ASD registered [6,87,88] as well as because the potential negative effects of EDCs have not been examined until recently. Equally, on several studies published after that date, the cohorts of children were actually born in the 1990s and early 2000s.

Inclusion criteria, based on PECO statement, were: (a) original articles; (b) observational (i.e., cohort, case-control and cross-sectional) studies; (c) only humans as study subjects without restriction of any demographic characteristics of the population; (d) exposure measured in women during pregnancy time period; (e) EDCs measured either (1) through questionnaires/interviews held with parents, (2) estimations provided by environmental agencies, or (3) analyses of biological samples; and, (f) the search was not restricted by language. Review articles, hypothesis papers, individual medical case studies, theses/dissertations, conference papers, and letters to the editor, as well as publications of animal models were excluded from this study.

The evaluation of the risk of bias on the retrieved studies for the domain “outcome assessment” was based on the clinical criteria of diagnosis of ASD (Diagnostic and Statistical Manual of Mental Disorders (DSM)) [89,90] or the International Classification of Diseases (ICD). It was also taken into account if the data came from self-reports, direct observational assessment by a qualified clinician, or from record-based diagnoses from public agencies, such as the California Dept. of Developmental Services (DDS) or the Autism and Developmental Disabilities Monitoring (ADDM) Network. Additionally, this domain was evaluated for the risk of bias depending on the tool used on these articles: (1) routine developmental surveillance, such as the Social Responsiveness Scale (SRS) [91,92], the Social Communication Questionnaire (SCQ), and the Korean-Child Behavior Checklist (K-CBCL) as measures for emotional and behavioral problems (non-specific ASD); (2) specifically screen for ASD, such as the Autism Behavior Checklist (ABC), Quantitative Checklist for Autism in Toddlers (Q-CHAT), Modified Checklist for Autism in Toddlers (M-CHAT), and The Autism Treatment Evaluation Checklist (ATEC); and, (3) definitive diagnostic confirmation using the Childhood Autism Rating Scale (CARS), Autism Diagnostic Interview-Revised (ADI-R) and Autism Diagnostic Observation Schedule-Generic (ADOS-G). Of the above, only the ADI-R and ADOS-G (conducted jointly) have been seen as ‘gold standard’ diagnostic instruments that are appropriate for use in ASD research protocols. At the present time, however, neither tool has been standardised against the DSM 5, the forthcoming ICD 11 revision, or the NIH RDoC system.

Additionally, the assessment of the methodological quality of each eligible paper was performed in accordance with the methods section checklist of the Strengthening the Reporting of Observational Studies in Epidemiology (STROBE) statement [93]. Thus, to assess the evidence provided by the included papers based on STROBE, we considered the following features: (a) sample size and degree of homogeneity of the group studied; (b) use of a control group and the appropriateness of that selection; (c) type of observational design; (d) nature and degree of exposure to EDCs; (e) selection of assessment criteria—including the quality of the ASD diagnosis and the instruments or methods used (analyses in biological fluids, environmental reports and questionnaires/interviews); and, (f) adjustment for confounding factors, such as pharmacological treatments provided or environmental factors that could affect this association.

### 2.4. Data Extraction

The process of selecting the articles to be included in this review was carried out in two steps. First, two different groups (SMB, CDV, AMS & MMSV, IPC, ALLG, SMR) from the research team independently assessed and screened the titles and abstracts of each potential study and collected descriptive information. Second, the studies that were selected in this first step were further examined by two members (SMB and MMSV) with expertise in epidemiology and the environmental health field. The resultant studies were compared to determine agreement for the search and inclusion criteria. The final overall quality and strength of the evidence was independently evaluated by each author. Finally, the evaluations were compared, the discrepancies discussed, and the final decisions were justified collectively.

## 3. Results

### 3.1. Characteristics of Studies

The search yielded 17 publications that met the inclusion criteria from a pool of 251 potential studies, and involved case-control and cohort study designs. The main differences among studies included were the sample size—from 30 to around 300,000 patients, age of children, and the measurement of the exposure to EDC. Regarding the data collection method of the studies, three (18%) used questionnaires/interviews, (Table 4), eight (47%) based on estimations provided by environmental government agencies (Table 5), and six (35%) used analytical methods and biological samples (Table 6). The majority of the studies adjusted the assessments for several potential confounders, such as maternal age, parental level of education, race/ethnicity, gender of child, household income, tobacco smoke status, and some measure of socio-economic status. Due to the limited number of studies involved as well as their different methodologies we could not perform a meta-analysis.

### 3.2. Internal Validity for Individual Studies

**Figure 2:**
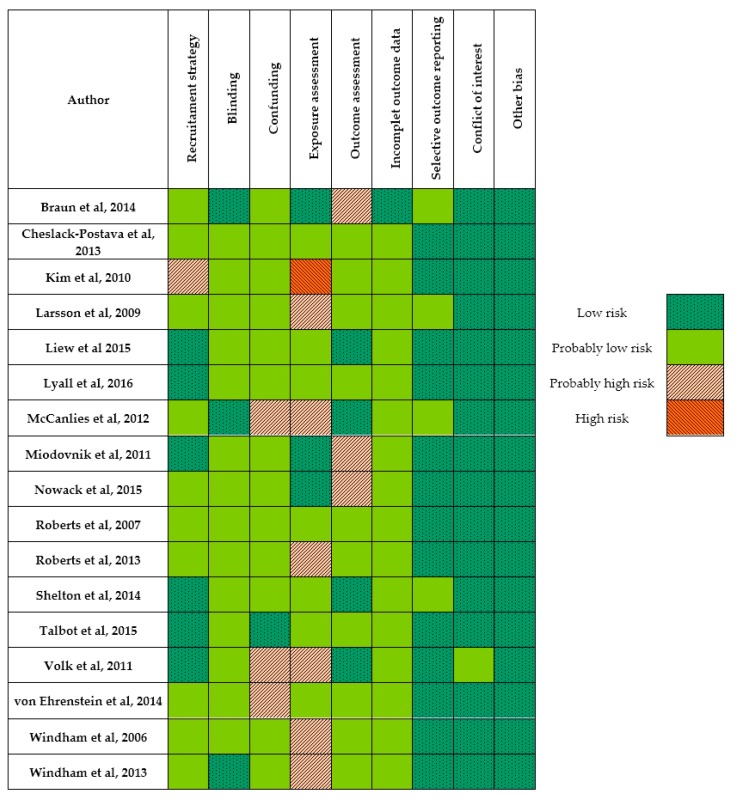
Summary of risk of bias assessment across individual studies. Review of authors’ judgments (low, probably low, probably high, and high risk) of bias for each risk of bias domain for each included study (*n* = 17).

We evaluated the internal validity (risk of systematic bias) in each study. Several studies presented validity problems, such as the no specification of inclusion criteria, the small sample size, the lack of a control group, as well as the failure to take into account phenotypic variability between individuals or to explore alternative explanations. In addition, errors in the data that were provided through questionnaires/interviews answered by parents with insufficient training or values being deliberately biased are likely. The studies that were ultimately rated as having “high” or “probably high” risk of exposure assessment bias used data from questionnaires/interviews, or estimated the exposure from the national and public data, such as the Toxic Release Inventory (TRI) or the US EPA National-scale Air Toxics Assessment (NATA). Likewise, well-known potential confounding variables were not taken into account in some of the studies. However, in the majority of domains other than those referring to exposure assessment and confounding factors, most studies were rated as “low” or “probably low” risk of bias (Figure 2).

### 3.3. Risk of Bias Exposure Assessment for Individual Studies

The exposure assessment risk of bias in those studies using interviews/questionnaires was “high” in Kim et al., (2010) (PBDEs, PCBs, BPA, and PCDD); and, “probably high” in Larsson et al., (2009) [94] (phtalates) and McCanlies et al., (2012) [95] (asphalt and solvents).

Among studies using NATA data, Robert et al., 2013 [96] (mercury, lead, nickel, methylene chloride, and diesel), Volk et al., 2011 [27] (traffic-related air pollution near a freeway), Windham et al., 2006 [97] (chlorinated solvents and heavy metals), and Windham et al., 2013 [98] (exhaust and combustion products and disinfectants) were assigned as “probably high” risk of exposure assessment bias. Likewise, Roberts et al., 2007 [99] (organochlorine pesticides), Shelton et al., 2014 [100] (organophosphorus pesticides [cholpyrifos], heavy metals, pyrethroids, styrene, and PHA), Talbott et al., 2015 [101] (styrene), and von Ehrenstein et al., 2014 [102] (1,3-butadiene, lead, benzene, toluene, ethyl-benzene, xylenes, formaldehyde, and chlorinated solvents) were ranked as “probably low” risk of exposure assessment bias.

Among studies based on laboratory analysis, Braun et al., 2014 [19] (phthalate metabolites, BPA, PCBs, organochlorine pesticides, brominated flame-retardants, and PFAS), Miodovnik et al., 2011 [103] (metabolites of the phthalates, and BPA), and Nowack et al., 2015 [104] (PCDD/Fs and PCBs) were classified as “low” risk of bias. Equally, Cheslack-Postava et al., 2013 [105] (PCBs, HCB, DDE, and PFAS) and Liew et al., 2015 [106] (PCBs and OCPs) were assigned as “probably low”.

### 3.4. Summary of Results

In general, we observed a trend towards positive effects (exposure to overall EDCs was associated with an increased risk of ASD). One of the primary reasons for rating up the quality of evidence is when a large enough magnitude of effect exists [107]. According to the GRADE working group [107] guidelines, the magnitude of effect that may increase the quality of evidence are as follows: large magnitude of effect (direct evidence, relative risk [RR], or odds ratio [OR] = 2–5 or RR/OR = 0.5–0.2 with no plausible confounders); very large magnitude of effect (RR/OR > 5 or RR/OR < 0.2 and no serious problems with risk of bias or precision with sufficiently narrow confidence intervals). The groups of EDC that reported consistently significant OR/RR of ASD > 2 were: “industrial chemical contaminants” (e.g., lacquers, asphalt, styrene and xylene), “exhaustion and combustion products”, “agricultural pesticides” (e.g., pyrethroids, organochlorines, and organophosphates), and “plastics” (bisphenol A). Those EDCs that reported OR/RR < 2 included “heavy metals” (cadmium, chromium, lead, nickel, and mercury) and “phthalates”. PFAS and DDT metabolites did not reach the statistical significance. However, there was a considerable risk of systematic bias due to the exposure assessment—with several studies rated as “high” or “probably high”—for many of these EDCs.

### 3.5. Quality of the Overall Body of Evidence

Based on our evaluation using the Navigation Guide criteria, we rated the initial quality of evidence across overall EDCs as “moderate”. The decisions leading to this rating are based primarily on the concern that many of the studies showed “high” or “probably high” exposure assessment risk of bias, mainly because of the exposure assessment methodology, which included: extrapolation of data from the amount of emissions to individual or community exposures, measuring exposure using varied metrics (i.e., environmental monitoring, emissions-based modeling, or occupation/work place as exposure estimation). Nevertheless, because of several EDCs shown OR or RR of ASD greater than 2, we did not need to degrade or upgrade the evidence, and therefore the initial “moderate” rating was retained.

### 3.6. Strength of the Overall Body of Evidence

Prenatal exposure to EDCs was associated with an increased risk of ASD development. However, this relationship is constrained, and then cannot be ruled out with reasonable confidence by such factors as: quality of the overall body of evidence, the direction of the effect, confidence in the effect, the number, or size of studies included. Based on the consistency of the findings across the studies, we concluded that the final overall strength of the evidence on a positive association between prenatal EDC exposure and offspring ASD development is “limited” (Table 7).

## 4. Discussion

We applied the Navigation Guide systematic review method to summarize the evidence of the association between prenatal EDCs exposure and later ASD development in children. To date, the Navigation Guide method has been used in few studies [108,109,110,111,112,113]. We believe however, that this methodology guarantees greater confidence, since it is a systematic, robust, and rigorous approach to research synthesis in the evaluation of evidence-based medicine and environmental health. Likewise, it is based in a pre-defined question and protocol, standardized and transparent documentation, including a comprehensive search strategy as well as more accurate assessment of “risk of bias” for individual studies.

This systematic review aimed to evaluate the quality individually in each of the studies assessing the potential higher odds of a future ASD of those children whose mother were exposed to EDC during pregnancy and summarize the existing evidence to date. In general, the studies found that those mothers with children that were diagnosed with ASD were more exposed to EDC during pregnancy. However, we found that the quality and strength of these studies were “moderate” and “limited”, respectively.

### 4.1. Limitations of the Review Process

This review process has some limitations. First, the review itself may be sensitive to publication bias and we might not have retrieved all of the relevant publications on the subject (e.g., studies that could have had repercussion in the conclusion of this review). To take into account the different methodologies used to measure the EDCs, we grouped the studies based on the method of the exposure assessment used. However, the wide variety of different EDCs addressed, the different times of exposure (first, second, or third trimester of pregnancy), as well as the differences in the methodology of the ASD across the studies are other limitations of this review. Although the variability in the diagnostic criteria among studies is representative of the time in which the study was conducted, it could affect the estimations. This suggests the need for a unified and globally accepted diagnostic criterion, i.e., the DSM-5.

In order to faithfully apply all of the steps recommended in the methodology of the Navigation Guide, human and non-human evidence should be integrated. This is, however, a preliminary study of a larger project that will also include a systematic review of the current experimental and animal evidence of the suggested relationship between the EDCs and autistic behavioral outcomes.

### 4.2. Overview of the Topic, Recommendations and Implications

The methodological limitations identified on retrieved studies were associated with a range of factors: the lack of a control group and/or clear definitions of inclusion criteria, very small sample sizes, groups being heterogeneous in terms of age or failure to control for phenotypic variability between individuals. There was also a risk of bias due to behavioral variables that were attributable to the memory of parents and other caregivers being distorted over time, or lack of adjustment for potential confounding variables (maternal age, maternal race, maternal education level, maternal BMI (body mass index), maternal smoking, maternal social status, family income, infant sex, gestational age, alcohol consumption, country of birth, delivery type, or birth weight, among others).

On the other hand, there are several reasons for which the evidence obtained from the studies included in this review may not be classified as “sufficient”: the time lapse between chemical exposure and ASD diagnosis; the difficulty of calculating real exposure levels; and, the lack of information on the effects of combined exposures, among others.

### 4.3. Biological Plausibility

Animal experiments allow us to assess the biologic plausibility of the associations that were observed in epidemiologic studies (e.g., the relationship between exposure to EDCs and ASD) and mechanisms of action have been inferred from animal and in vitro models. Animal studies have shown that these exposures generally result in decreased T4 levels and/or increased TSH levels. In rats, a relationship between PBDE exposure and learning and memory alteration has been observed and the chronic exposure of parent zebrafish to low doses of PBDE led to neurobehavioral changes in their offspring [114]. Gestational exposure to CPF (chlorpyrifos) in rats impairs neuronal differentiation, synaptogenesis, and gene expression, and it affects the cholinergic, serotoninergic, and dopaminergic neurotransmitters, in a sex-dimorphic fashion [115].

EDCs and their metabolites can interact with the endocrine molecular signaling system as ligands (agonist or antagonist or co-activator) of transcription factors, disrupting the normal neuro-physiological mechanisms [116].

For instance, thyroid hormones play a critical role in neurodevelopmental processes, such as neuronal growth, cell migration, synaptogenesis, and myelination. The foetus cannot produce Thyroid-stimulating hormone (TSH) before the second trimester of pregnancy and it is entirely dependent on maternal thyroid hormone. TSH regulates the synthesis and secretion of thyroid hormones, which in turn are involved in neurodevelopment. TSH levels can be moderated by the hypothalamus through the release of the thyrotropin releasing hormone (TRH) and interactions between the hypothalamus–pituitary–thyroid/gonadal axis can be inhibited or stimulated by exposure to chemical pollutants [71,72].

ASD is approximately four times more prevalent in males than in females. This difference indicates that sex hormones likely also play a role in the disorder [66]. The authors of the “extreme male brain theory” of autism observed elevated fetal steroid hormones (including testosterone, estradiol, progesterone, androstenedione, and cortisol, among others), linked to ASD in their children [67].

Prenatal exposures to EDCs may induce a variety of autistic features (ASD applies to a very heterogeneous group of people with different levels of ability and severity), through changes in gene expression, leading to altered hormonal signaling pathways [68,115]. For instance, DNA methylation [117], histone modifications [118], and altered microRNA expression [119,120] produce alterations in the metabolome, which affects neural pathways linked to behaviors that are associated with ASD. EDC-induced phenotypic changes have been linked to ASD-specific epigenetic changes [121,122].

## 5. Conclusions

This description of studies published to date aims to serve as a summary of the current available scientific evidence. The current limited epidemiological studies, the weak associations of the retrieved studies, and incomplete understanding of biological mechanisms precludes the establishment of a causal relationship. However, the ubiquitous presence of EDCs, their persistence and bioaccumulation, and the biologically plausibility highlight the need to carry out well-designed studies on the associations between EDC exposure during pregnancy and ASD during childhood. Future studies should overcome the limitations present in the studies conducted so far, and, for instance, be developed in larger datasets quantifying exposures using biomarkers and with validated instruments. Likewise, identify critical windows of vulnerability not only during embryo and fetal development, but also during infancy, early childhood, and adolescence is also required. Also, given the evidence that does exists, it is important to be aware of this risk that exposure to EDCs may pose and minimize its impact as much as possible. Prevention and early intervention should become the goal for all professionals involved, from environmental scientist to health professionals, such as obstetricians, gynecologists, midwives, pediatricians, GPs, neurologist, psychiatrist, psychologist, and occupational health professionals given the vulnerability of those involved and the possible long term effects of exposure.

## Figures and Tables

**Table 1:** Chemical groups and subgroups of substances with endocrine disrupting potencial (Brouwers et al.,2009).

Chemical Group	Subgroups	Description	Reported Endocrine Disrupting Effects
Polycyclic aromatic hydrocarbons	None	Formed by incomplete combustion of carbon-containing fuels.	Anti-estrogenic effects in vitro
Polychlorinated organic compounds	Polychlorinated biphenyls (PCBs) Dioxins, furans, polychlorinated naphthalene (PCN) Hexachlorobenzene (HCB) Octachlorostyrene (OCS)	Produced as by-products during waste incineration and industrial processes involving carbon and chlorine (eg., during metal, solvent or pesticide manufacturing) PCBs: until 1970s widely used as insulating and cooling fluids	PCBs, dioxins, furans, PCN: interfere with steroid synthesis through aryl hydrocarbon receptor binding; HCB: affects male and female fertility in animal studies; OCS: their metabolites possibly interfere with thyroid homeostasis through binding to plasma proteins
Pesticides	Organochlorines CarbamatesOrganophosphatesTributyltin Pyrethroids Other pesticides	Used in agriculture.Other purposes include wood preservation, anti-fouling, parasite treatment and public hygiene	Estrogenic or anti-androgenic effects in vitro, reproductive toxicity in animal models, and fertility or endocrine alterations in human studies
Phthalates	Di-2-ethylhexyl phthalate (DEHP), di-isononyl phthalate (DNP), di-n-hexyl phthalate (DHP) Benzylbutyl phthalate (BBP) Dibutylphthalate(DBP) Diethyl phthalate (DEP)	Many industrial applications:High molecular weight phthalates (DEHP, DNP, DHP) primarily used a plasticisers in polyvinyl chloride(PVC).Low molecular weight phthalates (BBP, DBP, DEP) used as solvents and plasticisers in cosmetics, adhesives, ink, dyes and plastic packaging	DEHP, DNP, DHP, BBP, DBP: affect the development of male reproductive organs in animal studies; DEP, DBP, BBP: suggested to interfere with male reproductive hormone levels in children
Organic solvents	Ethylene glycol ethers (EGEs) Styrene 5oluene Xylene Trichloroethylene (TCE) Perchloroethylene (PCE)	EGEs, toluene, xylene: widely used in, for example, paints, adhesives, thinners, lacquers and resinsStyrene: used for producing polystyrene plastics and resinsTCE, PCE: used for metal degreasing and other industrial cleaning purposes	EGEs: reproductive toxicity in animal studies and possibly associated with reduced fertility and menstrual length variability in women; Styrene: styrene dimers and trimers bind to estrogen receptors in vitro; Toluene, xylene, TCE: suggested to interfere with reproductive hormone levels in humans. PCE: dry cleaning has been associated with menstrual disorders, infertility and delayed conception in women
Bisphenol A	None	Used in the production of polycarbonate plastic and epoxy resins	Estrogenic effects according to in vitro and in vivo studies
Alkylphenolic compounds	Alkylphenolic ethoxylates (APEs) Alkylphenols (APs)	APEs: commonly used surfactants in, for example, detergents, pesticides and cosmetics; APs: primarily used to produce APEs	APE metabolites, which include APs and short chain APEs, interact with estrogen receptors in vitro
Flame retardants	Tetrabromobisphenol A (TBBPA) Hexabromocyclodecane (HBCD) Polybrominated disphenyl ethe r(PBDEs)	Widely used in the polymer industry, for example in the production of PVC, epoxy resins, polyesteand rubber	TBBPA, HBCD, PBDEs: interfere with thyroid hormone levels. TBBPA, PBDEs: possibly interfere with estrogen metabolism through estrogen sulfotransferase inhibition
Metals	Arsenic Cadmium Copper Lead Mercury	E.g., used in the electrical/electronics industry, for construction, in batteries, dyes, pesticides and dental amalgam, and as chemical intermediates	Arsenic: inhibits glucocorticoid gene transcription in vitro and thought to have similar effects on other steroid receptors. Cadmium, copper, lead, mercury: testicular toxicity in animal models or altered hormone levels and/or male subfertility in humans.
Miscellaneous	Benzophenones Parabens Siloxanes Phytoestrgens Pharmaceutical chemicals	Benzophenones: UV screens used in cosmetics and the plastic industry Parabens: widely used preservatives in cosmetics and the pharmaceutical industry Siloxanes: intermediates in the polymer industry and ingredients in personal care products and precision cleaning agents Soy and other plant products.	Benzophenones: bind to estrogen receptors in vitro and exert estrogenic effects in animal studies. Parabens: estrogenic activity in vitro and in animal Studies. Siloxanes: estrogenic and anti-estrogenic activity in animal studies

**Table 2:** Criteria for assigning quality and strength of evidence to observational studies (The Navigation Guide Systematic Review, Woodruff et al., 2011).

Risk of Bias ^a^	Quality of Evidence ^b^	Strength of Evidence ^b^
Domains: Recruitment strategy Blinding Confunding Exposure assessment Outcome assessment Incomplete outcome data Selective outcome reported Conflict of interest Other bias Evaluation: *Determined for each risk of bias domain* Low risk Probably low risk Probably high risk High risk	*Human evidence begins as moderate* Downgrade criteria *−1 or −2 according these factors:* Risk of bias across studies Inconsistency of results Indirectness of evidence Imprecision Publication bias Upgrade criteria *+1 or +2 according these factors:* Large magnitude of effect Dose response: Evidence of a gradient All plausible confounding would confirm negative results	*The final rating represent the level of certainty of toxicity.* Quality of body evidence: Direction of effect Confidence in effect Other compelling attributes of the data that may influence certainty Toxicity evidence rating Sufficient Limited Inadequate Lack of toxicity

^a^ Determined for each individual study; ^b^ Rated across all studies.

**Table 3:** Search strategy.

	Terms
#1	“Autism spectrum disorder” [MeSH] OR “Autistic disorder”[MeSH] OR “Child Development Disorders, Pervasive”[Mesh] OR “Child Behavior Disorders”[Mesh] OR “ autistic traits”
#2	“Environmental Exposure”[Mesh] OR “Endocrine Disruptors”[Mesh] OR “Pesticides”[Mesh] OR “Polychlorinated Biphenyls”Mesh] OR “Hydrocarbons, Chlorinated”[Mesh] OR “Dichlorodiphenyl Dichloroethylene”[Mesh] OR “DDT”[Mesh] OR “Hexachlorobenzene”[Mesh] OR “Flame Retardants”[Mesh] OR “Polybrominated Biphenyls”[Mesh] OR “Perfluorooctane sulfonic acid” [Supplementary Concept] OR “Bisphenol A” [Supplementary Concept] OR “Perfluorooctanoic acid” [Supplementary Concept]
#3	“pregnancy” OR “prenatal” OR “*in utero*”
#4	#1 AND #2 AND #3

**Table 4:** Interview and questionnaires to parents.

Author and Year	Study Population and Sample Size (*N*)	Study Design	Exposure	Outcome	Results
Kim et al., 2010	106 mothers of children with ADS and 324 mothers of typically developing (TD).children, and were recruited from special and elementary schools respectively in Seoul, Chungju, and Chuncheon, South Korea	Case-control study.	Self-reported exposure. Two questionnaires (knowledge/exposure) asking about the potential risk to EDCs. These questions regarding possible exposure to PBDEs, PCBs, BPA and PCDD were selected based on the guidelines provided by the study ‘Ministry of Environment for the Republic of Korea’	The Child Behavior Checklist Korean version (K-CBCL) was used to assess the diagnosis and severity of behavioral traits of ASD in children	The knowledge regarding the possible toxicity to EDCs, such as PBDEs, PCBs BPA, PCDD was significantly higher in cases than controls (t = 2.9, *p* = 0.001) and self-reported exposure was significantly higher in cases than controls (t = 5.6, *p* = 0.001)
Larsson et al., 2009	72 children (60 boys, 12 girls) with ASD in the county of Värmland, Sweden	Retrospective study based on the DBH longitudinal cohort study	Questionnaire asking for type of floor material used at home (PVC, wood, linoleum, etc.) as source of phthalates.	Parentally-reported ASD	ASD aOR 1.66 (95% CI: 1.02–2.7) for children with PVC floor at home in comparison with those with other floor material. Poor ventilation was also associated with ASD.
McCanlies et al., 2012	174 families: 93 children with ASD and 81 TD children born and living in California, and enrolled in the CHARGE study	Case-control study	Industrial-Hygienist Evaluation Exposures, i.e., occupational exposure to asphalt and several solvents including nickel, chromium, iron, aluminum, lead, toluene, xylene, phthalate, PCBs), and collected retrospectively.	ASD were assessed on the Autism Diagnostic Interview Revised (ADI-R) and the Autism Diagnostic Observation Schedules (ADOS)	Higher exposure (OR ≥ 2) to asphalt and solvents were observed among parents with ASD children compared with parents of TD children. But no significant associations after correcting

ASD: Autism Spectrum Disorder; TD: Tipically developing; ATEC: Autism Treatment Evaluation Checklist PBDEs: polybrominated diphenyl ethers; PCBs: polychlorinated biphenyls; BPA: bisphenol A; PCDD: polychlorinated dibenzo-p-dioxin; DBH: Dampness in buildings and Health; DSM-IV: Diagnostic and Statistical Manual of Mental Disorders, 4th edition; CHARGE: Childhood Autism Risks from Genetics and Environment study.

**Table 5:** Estimation of concentrations of endocrine disruptors provided by environmental agencies.

Author and Year	Study Population and Sample Size (*N*)	Study Design	Exposure	Outcome	Results
Roberts et al., 2007	Cases: 465 children with ASD. Controls: 6975 paired TD children Central Valley (California)	Case-control study.	Residential proximity of sources of agricultural pesticides: organochlorines, organophosphates, tTrifluralin)	Children with ASD were identified through electronic files of the California Department of Developmental Services according the Diagnostic and Statistical Manual of Mental Disorders (DSM IV-R)	In children of mothers living within 500 m of field sites (the fourth quartile vs. the lowest non-zero quartile of organochlorine poundage) to those with mothers not living near field sites the aOR was for ASD of 6.1 (95% CI: 2.4–15.3).
Roberts et al., 2013	Cases: 325 children with ASD (46 girls, 279 boys). Controls: 22,101 TD children. From all 50 U.S. states.	Case-control study from the Nurses’ Health Study II cohort	US EPA concentrations of several pollutants according to residency: Antimony, arsenic, cadmium, chromium, lead, manganese, mercury, nickel, all metals, diesel particulate, styrene, and methylene chloride.	ASD diagnosis validated by telephone administration of the Autism Diagnostic Interview–Revised (ADI-R) to 50 randomly selected case mothers	Comparing the higher quintile score and the lowest quintile Lead: aOR = 1.6; 95% CI: 1.1, 2.3 Manganese: aOR = 1.5; 95% CI: 1.1, 2.2 Mercury: aOR = 2; 95% CI: 1.2, 3. NickelL: aOR = 1.7; 95% CI: 1.1, 2.5 Cadmium: aOR = 1.5; 95% CI: 1.0, 2.1 Total metals: aOR = 1.5; 95% CI: 1.0, 2.3 Styrene: aOR = 1.4; 95% CI: 1.0, 2.1 Methylenechloride: aOR = 1.8; 95% CI: 1.2,2.8 Diesel particulate: aOR = 2; 95% CI: 1.0,4.0
Shelton et al., 2014	486 cases (children with ASD) and 316 controls (TD children) CHARGE study California	Case–control study	Proximity of homes to agricultural pesticides is used to estimate pesticide exposure using the Pesticide Use Report (PUR). Pesticides included are organophosphates, carbamates, pyrethroids, organochlorates and chlorpyrifos	Children are administered the Autism Diagnostic Observation Schedule (ADOS), combined with the ADI-R	Residential proximity (within 1.5 km) to agricultural pesticides it was compared with binary (1 = exposed vs. 0 = not exposed) indicators during pregnancy and his association with ASD Organophosphate pesticides: aOR = 2.07; (95% CI: 1.23, 3.50) Chlorpyrifos: aOR = 3.31; (95% CI: 1.48, 7.42) Pyrethroids: aOR 1.87; (95% CI: 1.02, 3.43)
Talbott et al., 2015	217 cases (children with ASD) and two different control groups: 1) 224 matched TD children and 2) 5,007 controls generated from a random sample using birth certificates (BC). Pennsylvania	Case–control study conducted by the EPA-NATA	Exposure to arsenic, chromium, methylene chloride, styrene, lead, cyanide, PAHs among other from ambient air pollution concentrations are estimated using modelled data from the 2005 NATA data.	ASD self-reported by family is diagnosed according to specific tests either such as ADOS or the Social Communication Questionnaire (SCQ)	Comparing fourth to first quartile of exposures: Styrene aOR 1.61 (95% CI = 1.08–2.38) Chromium aOR 1.60 (95% CI = 1.08–2.38). Methylene chloride aOR 1.41; 95% CI = 0.96–2.07) PAHs aOR 1.44; 95% CI = 0.98–2.11 Remaining compounds were not statistically significant.
Volk et al., 2011	Cases: 304 children with ASD. Controls: 259 TD children. California	Case-control study based on the CHARGE study.	Residential proximity to a freeway during pregnancy as a surrogate for air pollution (traffic-related pollutants)	The diagnosis of ASD was evaluated from both the ADOS and the ADI-R	Residential proximity (≤309 m) was compared to distance to the nearest freeway during the third trimester of pregnancy and was associated with ASD in offspring (aOR = 2.22; 95% CI: 1.16–4.42). No association with living close to other main roads during pregnancy and ASD.
von Ehrenstein et al., 2014	Cohort of children (*n* = 148,722) of which 768 were diagnosed with ASD. Los Angeles County, California	Observational cohort study	1,3-butadiene, lead, benzene, toluene, ethyl-benzene, xylenes, formaldehyde, and chlorinated solvents measured by community-based air-monitoring stationsin mothers residing at 5km from air-toxics during pregnancy.	ASD cases are identified through records maintained by the California Department of Developmental Services and diagnosed according the DSM IV-R	ASD increased risk per interquartile-range increase of exposures: 1,3-butadiene: aOR = 1.59; 95% CI: 1.18–2.15 Meta/para-xylene: aOR = 1.51; 95% CI: 1.26–1.82 Lead: aOR = 1.49; 95% CI = 1.23–1.81 Perchloroethylene: aOR = 1.40; 95% CI: 1.09–1.80 Formaldehyde: aOR = 1.34; 95% CI: 1.17–1.52
Windham et al., 2006	Cases: 284 children with ASD. Controls: 657 TD children. Born in 1994 and live in San Francisco. California	Case-control study	Exposure to 25 environmental pollutants is estimated by the US EPA according to place of residence.	The diagnosis of ASD is made by qualified medical professionals according to the criteria of DSM-IV	ASD risk in the upper quartiles of chemical concentrations compared with those below the median. Chlorinated solvents: Methylene chloride: aOR = 1.50; 95% CI: 1.06, 2.13 Trichloroethylene: aOR = 1.47; 95% CI: 1.03, 2.08 Vinyl chloride: aOR = 1.75; 95% CI: 1.25, 2.43 Metals: Cadmium: aOR 1.54; 95% CI: 1.08, 2.20 Mercury: aOR 1.92; 95% CI: 1.36, 2.71 Nickel: aOR 1.46; 95% CI: 1.04, 2.06 Other exposures were not associated with ASD
Windham et al., 2013	Parental occupation was obtained from birth certificates for 284 children with autism and 659 controls, born in 1994 in the San Francisco Bay Area (California)	Case-control study	Self-reported occupation and industry exposures are coded into eight chemical groups (exhaust/combustion products, disinfectants, metals, pesticides, solvents, cooling fluids, and auto paint)	Autism cases are identified according the DSM-IV by qualified medical professionals	Mothers of children with ASD had a higher probability (aOR = 2.3; 95% CI: 1.3, 4.2) of working in occupations considered exposed compared to mothers of controls (non-exposed). The exposure categories of the greatest frequency among case mothers were exhaust and combustion products (aOR 12.0; 95% CI: 1.4, 104.6) and disinfectants (aOR 4.0; 95% CI: 1.4, 12.0).

TD: typically develop; EPA-NATA: US Environmental Protection Agency National-Scale Air Toxics Assessment; ADOS: Autism Diagnostic Observation Schedule; ADI-R: Autism Diagnostic Interview, Revised.

**Table 6:** Analysis of the concentrations of endocrine disruptors in biological samples.

Author and Year	Study Population and Sample Size (*N*)	Study Design	Exposure	Outcome	Results
Braun et al., 2014	175 pregnant women ≥18 year from Cincinnati.	Observational study with the prospective birth cohort HOME	8 phthalate metabolites, BPA, 25 PCBs, 6 organochlorine pesticides, 8 brominated flame retardants and 4 PFAS in maternal serum or urine samples taken at gestation weeks 16–26.	Mothers completed the SRS questionnaire when children were 4–5 years old to evaluate autistic behavior	*Trans*-nonachlor and PBDE-28 were associated with autistic behaviors, β = 4.1; 95% CI: 0.8–7.3 and β = 2.5; 95% CI: 0.6–5.6, respectively. Weak associations (not reaching the statistically significance) were observed for PCB-178 (β = −3.0; 95% CI: −6.3, 0.2), β-HCH (β = −3.3; 95% CI: −6.1, −0.5), PBDE-85 (β = −3.2; 95% CI: −5.9, −0.5) and PFOA (β = −2.0; 95% CI: −4.4, 0.4).
Cheslack-Postava et al., 2013	Cases: 75 children with ASD. Controls: 75 TD children. Finland	Nested case-control pilot study in the Finnish Maternity Cohort	It is measured different PCB congeners, PBDE, HCB, DDT, and its metabolite (DDE) in maternal serum samples taken during pregnancy.	ASD in children validated by the ADI-R.	No significant association with ASD was found for any compound. The aOR of ASD in the >90th of exposure was compared to the lower end of the control distributions: PCBs aOR 1.91 (95% CI: 0.57, 6.39) HCB aOR 0.89 (95% CI: 0.28, 2.76) DDE aOR 1.79 (95% CI: 0.51, 6.21)
Liew et al., 2015	220 cases (children with ASD), and 550 TD children (controls) are selected from the Danish National Birth Cohort, Denmark	Nested case-control study.	Six PFASs measured in maternal plasma collected in early or mid-pregnancy	All diagnoses are based on ICD-10, code F84.0	No associations were observed for any of the PFAS assessed in relation to ASD.
Lyall et al., 2017	Cases: 545 children with ASD Controls: 418 TD children. California	Population-based case–control study	Concentrations of 11 PCB congeners and 2 OCPs measured in banked second-trimester serum samples and was compared between both groups	The diagnosis of ASD based on DSM-IV-TR criteria	OCPs were no associated with ASD and only 2 PCB congeners showed significant association. Comparing highest with the first quartile of PCBs: the OR of ASD were PCB138/158: aOR = 1.79 (95% CI: 1.10, 2.92) and PCB153: aOR = 1.82 (95% CI: 1.10, 3.02).
Miodovnik et al., 2011	137 mothers and their children born at Hospital Mount Sinai. New York City	Observational prospective cohort study.	Concentration of 10 metabolites of phthalates and BPA of maternal urine samples taken during the third trimester of pregnancy.	Mothers completed the SRS for detecting and measuring the severity of autistic behavior.	ΣLMWP β 1.53; 95% CI: 0.25–2.8 MEP β = 1.38; 95% CI: 0.23,2.53
Nowack et al., 2015	Out of 133 invited parents, 100 filled out the questionnaire SRS (*N* = 100) Duisburg, (Germany)	Observational cohort study	Concentrations of PCDD/Fs and PCBs measured in maternal whole blood samples during pregnancy.	Diagnosis and measurement of autistic behavior by the SRS.	Overall PCDD/Fs and PCBs were negatively associated with autistic behavior, PCDD/Fs: β = −6.66 (95% CI: −11.88, 1.44. *p* < 0.05); PCB: β = −3.99 (95% CI:−8.61, 0.64. *p* = 0.09).

β-HCH: β-hexachlorocyclohexane. DDT: dichlorodiphenyltrichloroethane, DDE: dichlorodiphenyldichloroethylene.; BDE: tetrabromodiphenylether; HCB: hexachlorobenzene; HMWP: High molecular weight Phthalate. LMWP: Low-Molecular-Weight Phthalates. ICD-10: International Classification of Diseases, 10th Revision. MMP: Monomethyl Phthalate. MEP: Monoethyl Phthalate. MBP: Monobutyl Phthalate. MiBP: Mono-iso-butyl Phthalate PFAS: perfluoroalkyl substances; PCB: Polychlorinated biphenyls; OCPs: organochlorine pesticides; PCDD/Fs: Polychlorinated dibenzo-p-dioxins and dibenzofurans. PBDE: polybrominated diphenyl ether; PFOA: perfluorooctanoate. HOME: Health Outcomes and Measures of the Environment. SRS: Social Responsiveness Scale. DSM-IV-TR: Diagnostic and Statistical Manual of Mental Disorders, 4th Edition, Text Revision.

**Table 7:** Quality and strength of evidence (Woodruff et al., 2011).

Factor		Explanation
	Downgrades	
Risk of bias	0 to −1	Based on the high/probably high risk of bias across the studies, mostly driven by the exposure assessment methods and the outcome evaluation (DSM, ICD-10, SRS, ADOS, ADI-R). The lack of specificity across different types of EDCs was of special concern.
Indirectness	0 to −1	Based on the adequate assessment of the exposure at individual level. There is a lack of individual EDC (or metabolites) and/or the exposures are not directly measured; for instance, Larsson et al. (2009) uses the floor material, McConlies et al. (2012) the occupational exposure and Volk et al. (2011) the residential proximity as a indicators of EDC.
Inconsistency	0	With few exceptions [Liew et al. (2015), Cheslack-Postava et al. (2013), and Mc Canlies et al. (2012)] results across studies are generally consistent in the magnitude and direction. Although most of the studies showed positive EDC-ASD associations, the magnitude of the effect was small and the statistical significance was not reached in many of them.
Imprecision	0	We judged that the CIs for ASD risk were considered as being excessively large.
Publication bias	0	There was no reason to suspect of publication bias. The search was comprehensive, and the most studies were generally consistent among their findings.
	Upgrades	
Magnitude of effect	0 to +1	Most of the studies found slight effects (i.e., OR < 2). However, several studies showed greater evidence of risk of ASD (RR or OR > 2)
Dose-response	0	The authors considered that there was some evidence of a dose-response relationship.
Confounding	0	We did not find evidence that possible residual confounding influenced results. In the studies retrieved potential confounders and effect modifiers they were examined including population characteristics such as race and ethnicity distribution, whether the tract was urban or rural, level of education, age of participants, percentage of the population below the poverty line, and median household income, among other.
Overall quality of evidence	Moderate	We judge that the results obtained from the retrieved studies not change the quality of the initial evidence
Overall strength of evidence	Limited	Although there is a trend of a positive association between the prenatal exposure to certain EDCs and following risk of ASD in the offspring, because the limitations present in the available studies so far, any conclusion can be drawn.
